# Supplementary material for: Weakened western Indian Ocean dominance on Antarctic sea ice variability in a changing climate
Source: Nat Commun. 2024 Apr 16;15:3261. doi: 10.1038/s41467-024-47655-0 (PMC11021451; doi:10.1038/s41467-024-47655-0)
Supplement: Supplementary file 1 — Supplementary Information [file 41467_2024_47655_MOESM1_ESM.pdf]

Supplementary Information for

Weakened western Indian Ocean dominance on Antarctic sea ice variability in a changing climate

Li Zhang[1,2*+], Xuya Ren[1,2*], Wenju Cai[1,2,3+], Xichen Li[4] and Lixin Wu[1,2]

1. Frontiers Science Center for Deep Ocean Multispheres and Earth System and Key Laboratory of Physical Oceanography/Academy of the Future Ocean, Ocean University of China, Qingdao, China
2. Laoshan Laboratory, Qingdao, China
3. CSIRO Oceans and Atmosphere Flagship, Aspendale, Victoria 3195, Australia
4. Institute of Atmospheric Physics, Chinese Academy of Sciences, Beijing 100029, China

*These authors contributed equally to this work

[+]Correspondence to: Li Zhang (zhangli@ouc.edu.cn) and Wenju Cai (Wenju.Cai@csiro.au)

This supplementary information includes Figures S1-S16.

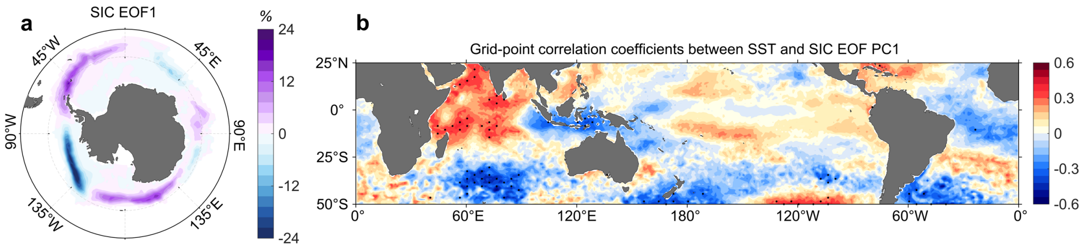

Supplementary Fig. 1 | Correlations of tropical SST anomalies with the Antarctic SIC modes. a Spatial pattern of Antarctic (60°S–90°S) SIC (%) EOF1 for the ASO 1979–2020 period. **b** Correlation between SIC EOF PC1 and each grid SST time series (dimensionless unit). Stippling indicates the 95% confidence level based on the *t*-test. Source data are provided as a Source Data file.

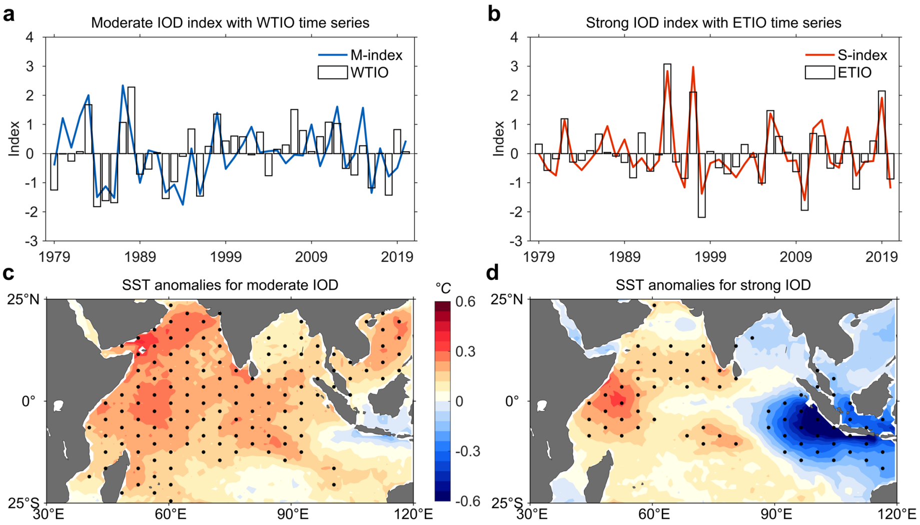

Supplementary Fig. 2 | Moderate and strong positive IOD. a Normalized time series of moderate IOD (M-index) and WTIO (i.e., Fig. 1b, red bar). **b** Normalized time series of strong IOD (S-index) and ETIO (i.e., Fig. 1b, red line). **c** Regression of SST (°C) onto the normalized M-index for the ASO 1979–2020 period. **d** Same as **c**, but onto the normalized S-index. Stippling indicates the 95% confidence level based on the *t*-test. See "Strong positive IOD and moderate positive IOD" in Methods. Source data are provided as a Source Data file.

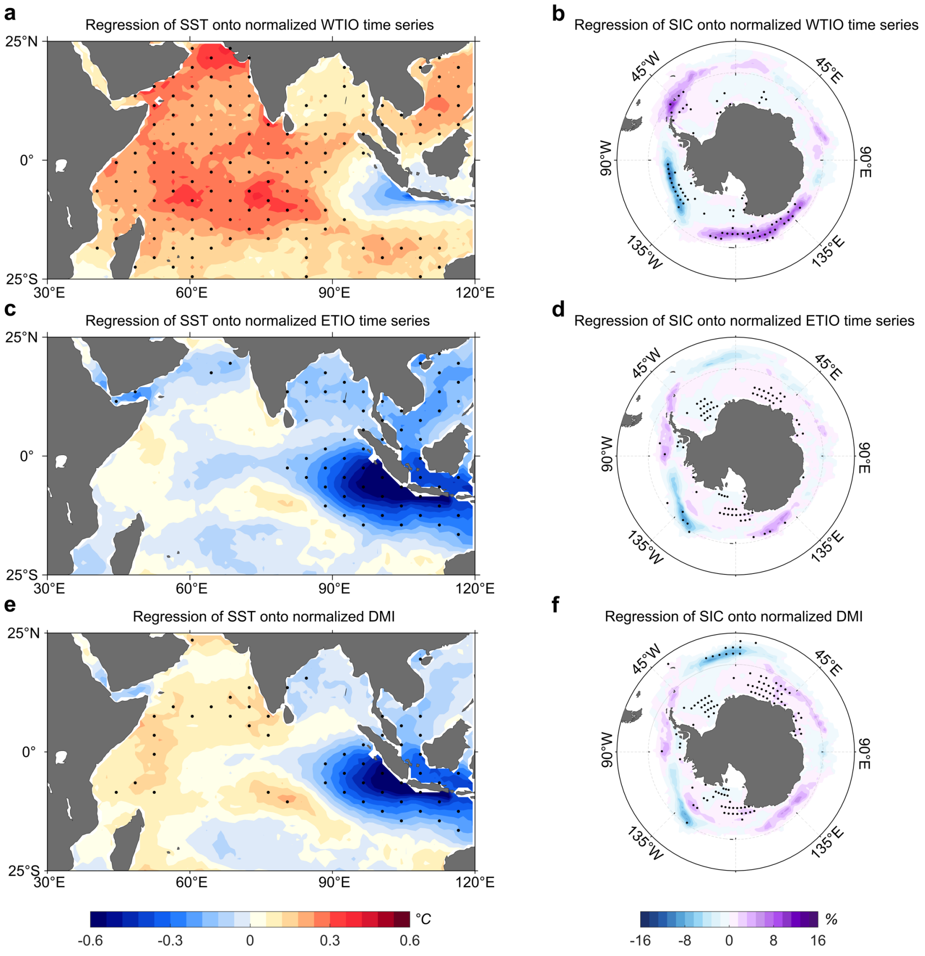

Supplementary Fig. 3 | Response of Antarctic SIC to ETIO SST anomalies and DMI. a, b Regressions of **a** SST (°C) and **b** SIC anomalies (%) onto the normalized WTIO time series for the ASO 1979–2020 period. **c, d** Same as **a, b**, but onto the normalized ETIO time series. **e, f** Same as **a, b**, but onto the normalized DMI. Stippling indicates the 95% confidence level based on the *t*-test. Source data are provided as a Source Data file.

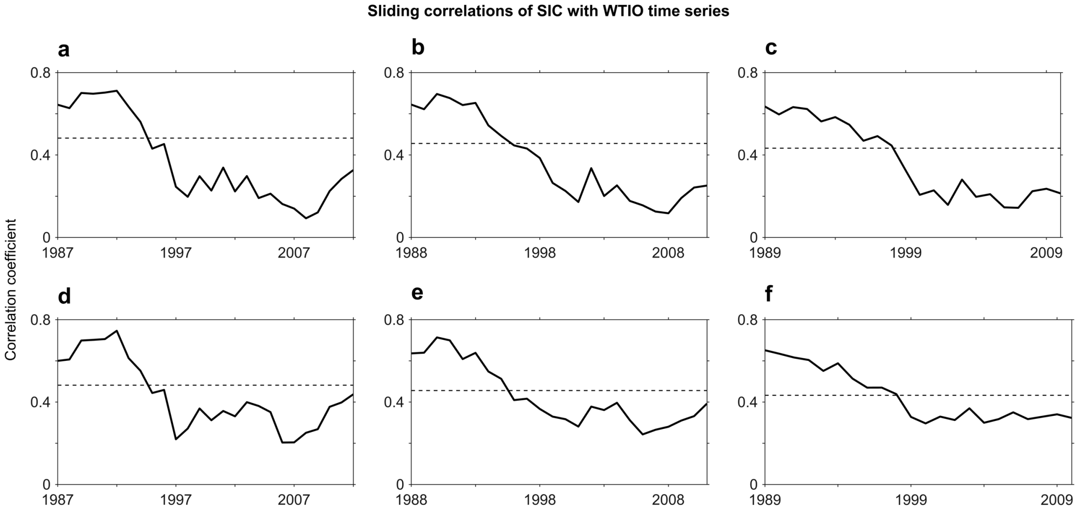

Supplementary Fig. 4 | Sliding correlation of the WTIO and Antarctic SIC. a-c 17-year, 19-year, and 21-year sliding correlation coefficients between the WTIO time series and Antarctic SIC EOF PC1 for the ASO 1979–2020 period. **d-f** Same as **a-c**, but for the September-October-November 1979–2020 period. The x-axis indicates the center year in the sliding window. See "Sliding correlation and sliding standard deviation" in Methods. Dashed lines indicate the 95% confidence level based on the *t*-test. Source data are provided as a Source Data file.

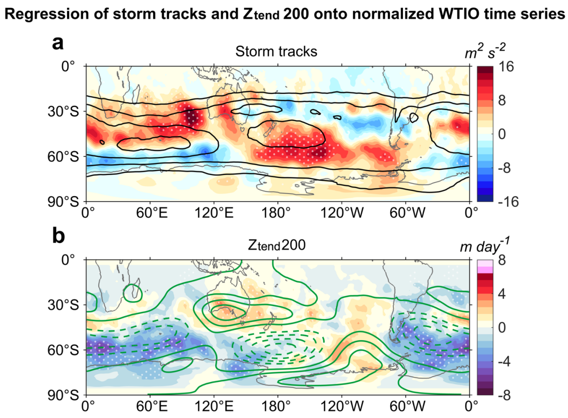

Supplementary Fig. 5 | Responses of synoptic-eddies feedback. a, b Regressions of **a** 200 hPa meridional wind variance (shading; m^2 s^{-2}; see "Meridional wind speed variance" in Methods; contours indicate climatology with interval 30 m^2 s^{-2}), **b** 200 hPa Z_{tend} induced by the sum of transient eddy heat flux and eddy vorticity flux (shading; m day^{-1}; see "Eddy-induced geopotential height tendency" in Methods), and Z200 (i.e., shading in Fig. 3b; green contours; positive solid and negative dashed; zero line omitted; starts from +/– 8 m and interval +/– 8 m) onto the normalized WTIO time series for the pre-1999 period. Stippling indicates the 95% confidence level based on the *t*-test. Source data are provided as a Source Data file.

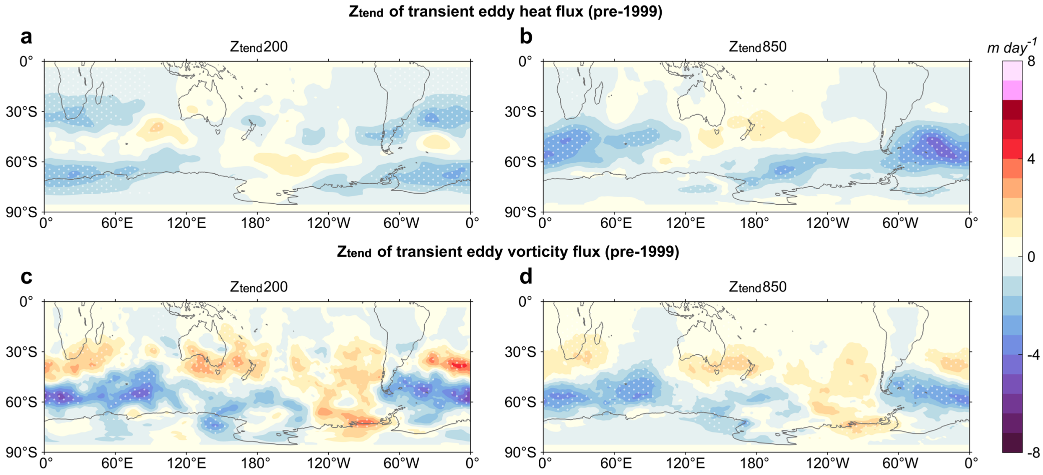

Supplementary Fig. 6 | Responses of geopotential tendency to transient eddy heat flux and transient eddy vorticity flux. a, b Regression of Z_{tend} (m day^{-1}) induced by transient eddy heat flux at **a** 200 hPa and **b** 850 hPa onto the normalized WTIO time series for the pre-1999 period. **c, d** Same as **a, b**, but by transient eddy vorticity flux. Stippling indicates the 95% confidence level based on the *t*-test. Source data are provided as a Source Data file.

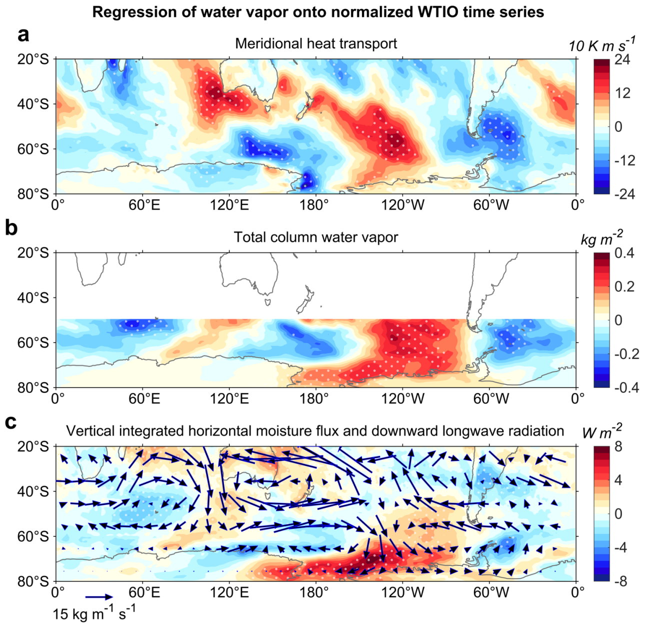

Supplementary Fig. 7 | Responses of water vapor around the Antarctic to WTIO SST anomalies. a-c Regressions of **a** meridional heat transport (K m s^{-1}), **b** total column water vapor (kg m^{-2}), **c** vertical integrated horizontal moisture flux (vectors; kg m^{-1} s^{-1}), and downward longwave radiation (shading; W m^{-2}) onto the normalized WTIO time series for the pre-1999 period. See "Meridional heat transport and vertically integrated horizontal moisture flux" in Methods. Stippling indicates the 95% confidence level based on the *t*-test. Source data are provided as a Source Data file.

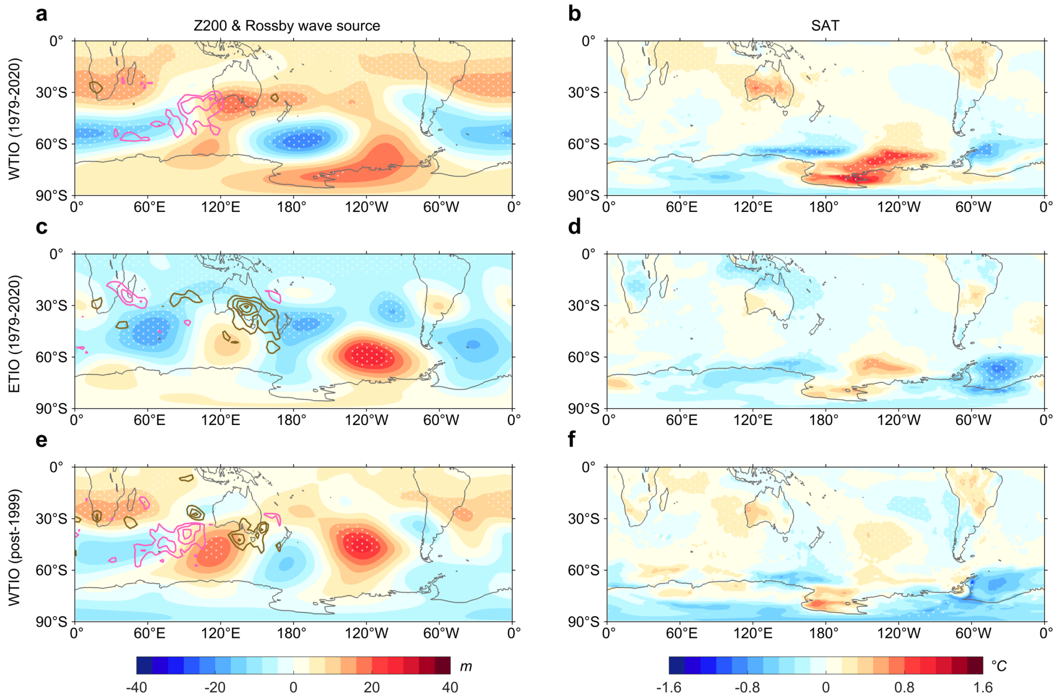

Supplementary Fig. 8 | Distinct teleconnections associated with the WTIO and ETIO SST anomalies. a, b Regressions of **a** Z200 (shading; m), Rossby wave source (contours; positive pink and negative brown; zero line omitted; starts from +/– 1.8×10^{-11} s^{-2} and interval +/– 1.2×10^{-11} s^{-2}), and **b** SAT (°C) onto the normalized WTIO time series for the ASO 1979–2020 period. **c**, **d** Same as **a, b**, but onto the normalized ETIO time series. **e, f** Same as **a, b**, but for the post-1999 period. Stippling indicates the 95% confidence level based on the *t*-test. Source data are provided as a Source Data file.

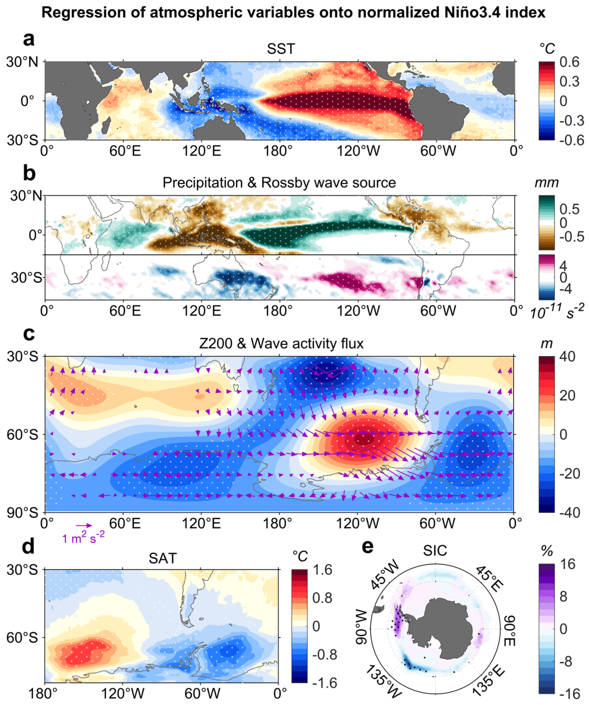

Supplementary Fig. 9 | ENSO related teleconnection. a-e Regressions of **a** SST (°C), **b** convective precipitation (mm; 15°S–30°N), Rossby wave source (10^{-8} s^{-2}; 45°S–15°S), **c** Z200 (shading; m), wave activity flux (vectors; m^2 s^{-2}), **d** SAT (°C), and **e** SIC (%) onto the normalized Niño 3.4 index for the ASO 1979–2020 period. Stippling indicates the 95% confidence level based on the *t*-test. Source data are provided as a Source Data file.

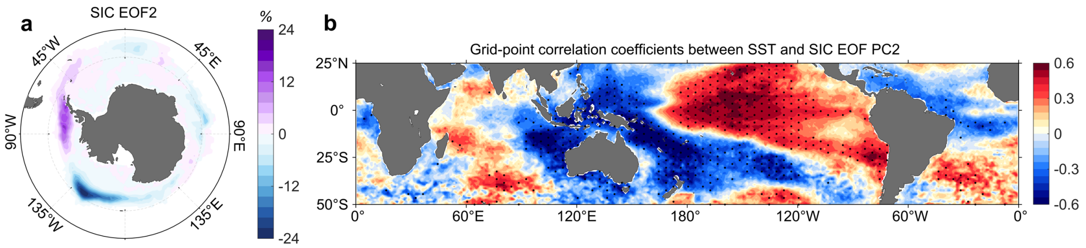

Supplementary Fig. 10 | Correlations of tropical SST anomalies with the Antarctic SIC modes. a Spatial pattern of Antarctic (60°S–90°S) SIC (%) EOF2 for the ASO 1979–2020 period. **b** Correlation between SIC EOF PC2 and each grid SST time series (dimensionless unit). Stippling indicates the 95% confidence level based on the *t*-test. Source data are provided as a Source Data file.

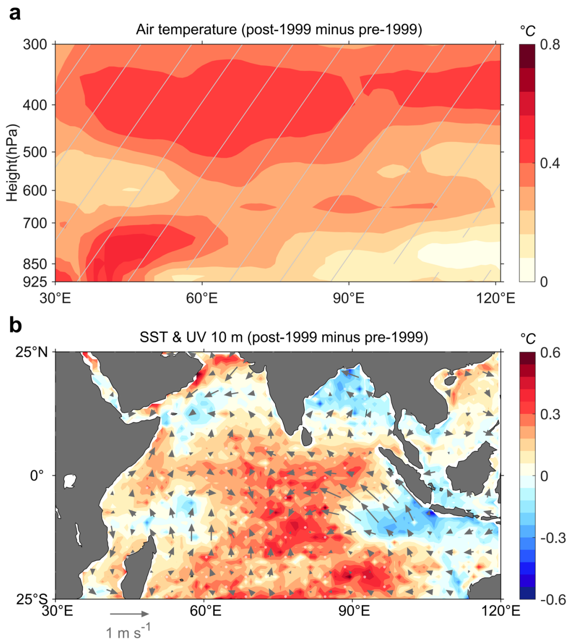

Supplementary Fig. 11 | Mean state change in the tropical Indian Ocean. a, b Climate difference of **a** tropical (10°S–10°N) air temperature, **b** SST (shading; °C), and 10 m wind (vectors; m s^{-1}) between the post-1999 and pre-1999 periods. Gray line in **a** and stippling in **b** indicate the 95% confidence level based on the two-tailed *t*-test. Source data are provided as a Source Data file.

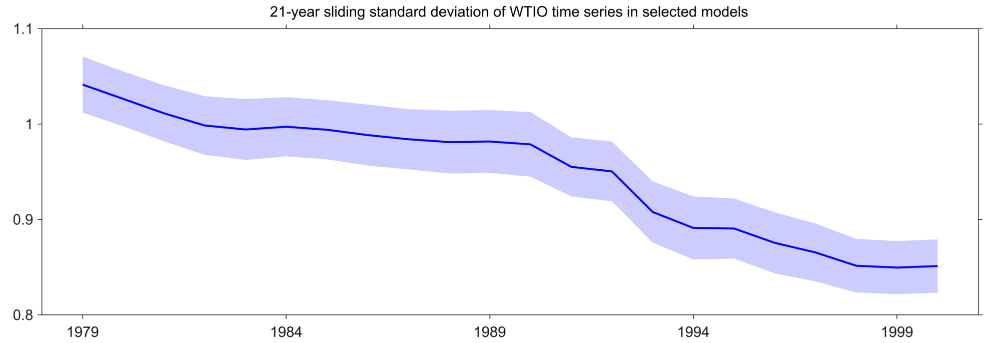

Supplementary Fig. 12 | 21-year sliding standard deviation of WTIO time series in CMIP6. Solid blue line and shading indicate selected multi-model mean (i.e., a total of 31 out of the 45 models without gray shading in Fig. 5a) and 1.0 standard deviation of a total of 10,000 inter-realizations based on a bootstrap method for the 1979–2020 period, respectively. The x-axis indicates the starting year in the sliding window. The decline trend is –0.093 standard deviation decade^{-1}. Source data are provided as a Source Data file.

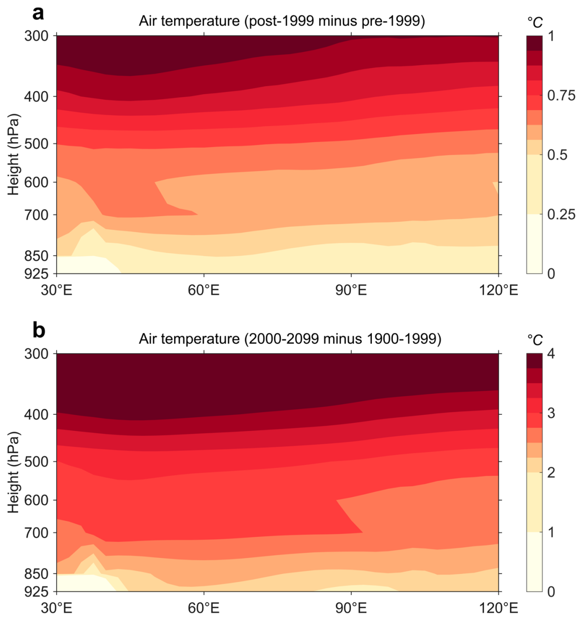

Supplementary Fig. 13 | Mean state change of air temperature. a Climate difference of tropical (5°S–5°N) air temperature (°C) between the post-1999 and pre-1999 periods, using 32 models with available geopotential height (i.e., models indicated in purple in Fig. 5). **b** Same as **a**, but between the future climate and the present-day. Source data are provided as a Source Data file.

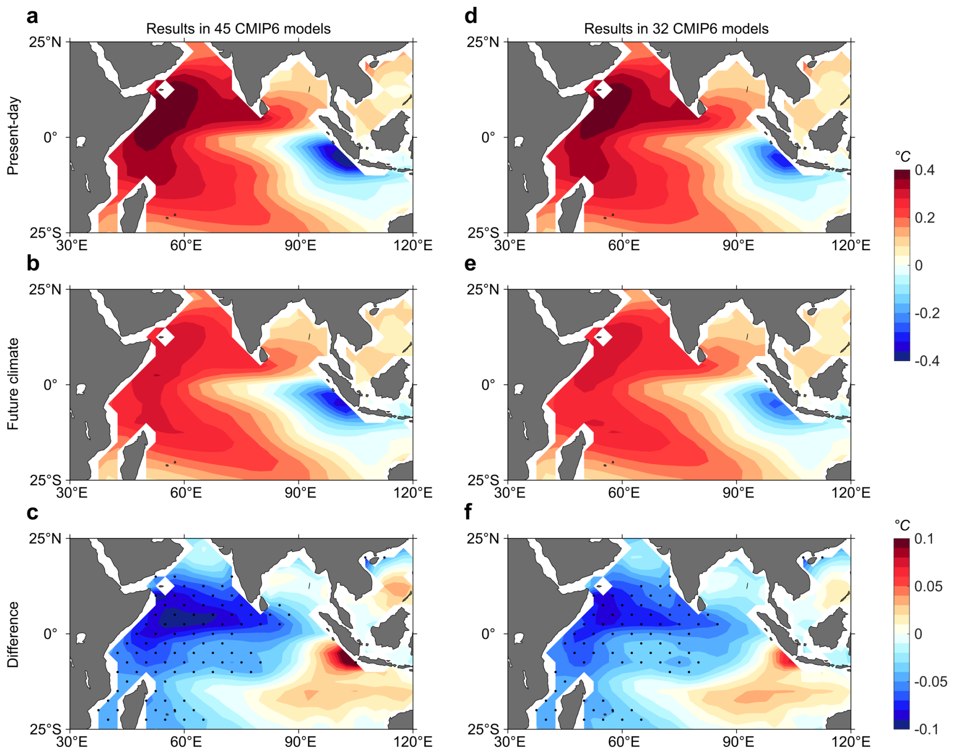

Supplementary Fig. 14 | Multi-model mean pattern of WTIO SST anomalies. a Multi-model mean regression of SST (°C) onto the normalized WTIO time series for the present-day. **b** Same as **a**, but for the future climate. **c** Difference of multi-model mean regression result between the future climate and the present-day (°C). **d-f** Same as **a-c**, but using 32 models with available geopotential height (i.e., models indicated in purple in Fig. 5). Stippling indicates the 95% confidence level based on the two-tailed *t*-test. Source data are provided as a Source Data file.

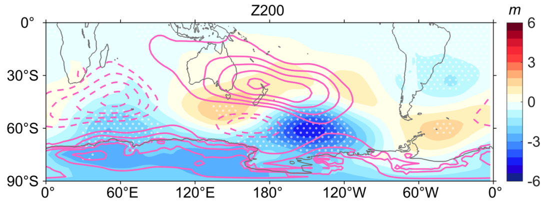

Supplementary Fig. 15 | Multi-model mean pattern of Z850 related to the WTIO. Z850 difference of multi-model mean regression result between the future climate and the present-day (shading; m), and the result for the present-day (contours; positive solid and negative dashed; zero line omitted; interval 0.5 m) without the influence of the tropical Pacific. Stippling indicates the 95% confidence level based on the two-tailed *t*-test. Source data are provided as a Source Data file.

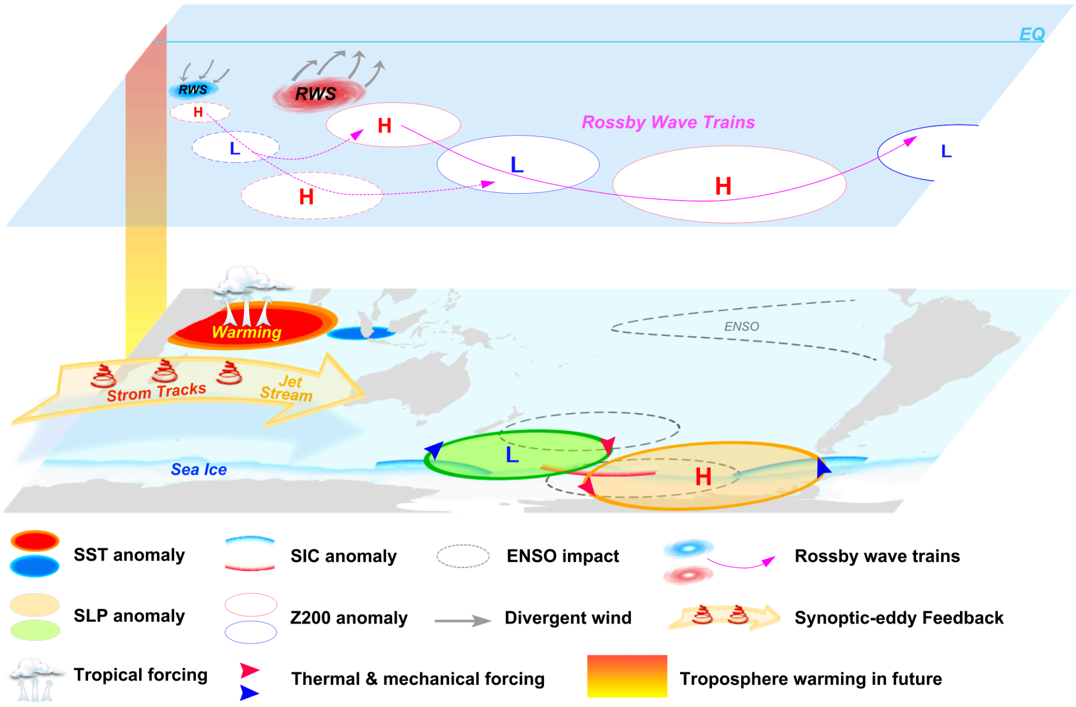

Supplementary Fig. 16 | Dominant atmospheric teleconnection associated with western Indian Ocean influence on Antarctic sea ice variability. Highlighted in the schematic are responses in the surface and upper troposphere. Warm WTIO SST anomalies (red shading) lead to changes in tropical convection (cloud with white arrows) driving anomalous divergent flow (gray arrows) in the upper troposphere. The divergent flow, in turn, excites Rossby wave sources (red and light blue vortices) in the subtropics and forces Rossby wave trains (pink lines) curve poleward and eastward towards Antarctica with alternating centers of high- and low-pressure anomalies (red and blue circles). The barotropic atmospheric response in SLP (green and orange shadings) alters Antarctic sea ice (red and light blue bold lines) through thermal advection and wind-driven drift (red and blue arrows). The pattern is distinctively different from that ENSO-induced (gray dashed line), and is reinforced by a synoptic-eddy feedback {Jet stream (orange arrow) with storm tracks (red helixes)}. This WTIO dominance of the Indo-Pacific influence on Antarctic sea ice has been weakening in line with projected change in the IOD by climate models (gradient orange shading).
